# Potential chimeric peptides to block the SARS-CoV-2 spike receptor-binding domain

**DOI:** 10.12688/f1000research.24074.1

**Published:** 2020-06-09

**Authors:** Debmalya Barh, Sandeep Tiwari, Bruno Silva Andrade, Marta Giovanetti, Eduardo Almeida Costa, Ranjith Kumavath, Preetam Ghosh, Aristóteles Góes-Neto, Luiz Carlos Junior Alcantara, Vasco Azevedo

**Affiliations:** 1 Centre for Genomics and Applied Gene Technology, Institute of Integrative Omics and Applied Biotechnology (IIOAB), Nonakuri, Purba Medinipur, WB, India; 2 Laboratório de Genética Celular e Molecular, Departamento de Biologia Geral, Instituto de Ciências Biológicas, Universidade Federal de Minas Gerais (UFMG), Belo Horizonte, Minas Gerais, Brazil; 3 Laboratório de Bioinformática e Química Computacional, Departamento de Ciências Biológicas, Universidade Estadual do Sudoeste da Bahia (UESB), Jequié, Bahia, Brazil; 4 Laboratório de Flavivírus, Instituto Oswaldo Cruz, Fundação Oswaldo Cruz, Rio de Janeiro, Brazil; 5 Núcleo de Biologia Computacional e Gestão de Informações Biotecnológicas (NBCGIB), Universidade Estadual de Santa Cruz (UESC), Km 16, Salobrinho, Ilhéus, Bahia, CEP 45662-900, Brazil; 6 Department of Genomic Science, School of Biological Sciences, University of Kerala, Tejaswini Hills, Periya P.O, Kasaragod, Kerala, 671316, India; 7 Department of Computer Science, Virginia Commonwealth University, Richmond, VA, 23284, USA; 8 Laboratório de Biologia Molecular e Computacional de Fungos, Departamento de Microbiologia, Instituto de Ciências Biológicas, Universidade Federal de Minas Gerais (UFMG), Belo Horizonte, Minas Gerais, Brazil

**Keywords:** Antiviral peptides, COVID-19, SARS-CoV-2, nCoV-19, peptide design, ACE2, Spike protein

## Abstract

**Background:** There are no known medicines or vaccines to control the COVID-19 pandemic caused by SARS-CoV-2 (nCoV). Antiviral peptides are superior to conventional drugs and may also be effective against COVID-19. Hence, we investigated the SARS-CoV-2 Spike receptor-binding domain (nCoV-RBD) that interacts with hACE2 for viral attachment and entry.

**Methods:** Three strategies and bioinformatics approaches were employed to design potential nCoV-RBD - hACE2 interaction-blocking peptides that may restrict viral attachment and entry. Firstly, the key residues interacting with nCoV-RBD - hACE2 are identified and hACE2 sequence-based peptides are designed. Second, peptides from five antibacterial peptide databases that block nCoV-RBD are identified; finally, a chimeric peptide design approach is used to design peptides that can bind to key nCoV-RBD residues. The final peptides are selected based on their physiochemical properties, numbers and positions of key residues binding, binding energy, and antiviral properties.

**Results: **We found that: (i) three amino acid stretches in hACE2 interact with nCoV-RBD; (ii) effective peptides must bind to three key positions of nCoV-RBD (Gly485/Phe486/Asn487, Gln493, and Gln498/Thr500/Asn501); (iii) Phe486, Gln493, and Asn501 are critical residues; (iv) AC20 and AC23 derived from hACE2 may block two key critical positions; (iv) DBP6 identified from databases can block the three sites of the nCoV-RBD and interacts with one critical position, Gln498; (v) seven chimeric peptides were considered promising, among which cnCoVP-3, cnCoVP-4, and cnCoVP-7 are the top three; and (vi) cnCoVP-4 meets all the criteria and is the best peptide.

**Conclusions:** To conclude, using three different bioinformatics approaches, we identified 17 peptides that can potentially bind to the nCoV-RBD that interacts with hACE2. Binding these peptides to nCoV-RBD may potentially inhibit the virus to access hACE2 and thereby may prevent the infection. Out of 17, 10 peptides have promising potential and need further experimental validation.

## Introduction

The world is currently experiencing the severe coronavirus disease 2019 (COVID-19) pandemic caused by SARS-CoV-2 or novel corona virus (nCoV) that originated from the Wuhan city, China ^1, 2^, and spread across the world. So far, two million people have been infected and more than 120,000 deaths are recorded across the globe. The death rate is 3–20% depending on the countries and the most affected countries are the USA, Italy, Spain, the UK and France, which have each recorded more than 10,000 deaths within a couple of weeks ( WHO COVID-2019 situation reports). SARS-CoV-2 is highly contagious in humans and so far no medicine or vaccine has been developed to tackle the virus, making it impossible to control its spread across the globe ^3^. Although drugs like hydroxychloroquine, remdesivir, and lopinavir ^4^ are currently being suggested to treat COVID-19 infection, there is no clinical study so far to prove their efficacy in treating these patients. Therefore, currently there is a global search for appropriate drug and vaccine candidates against SARS-CoV-2.

SARS-CoV-2 has shown 80% genome identity with SARS-CoV, which is the causal agent of severe acute respiratory syndrome (SARS) seen in 2002–2003 ^5^. SARS-CoV binds to the human angiotensin-converting enzyme 2 (hACE2) receptor through its Spike protein (S) to enter into the host cell ^6^, and it is now reported that SARS-CoV-2 also binds to ACE2 to transmit its genetic material to human cells ^7– 9^. Therefore, blocking the Spike protein of SARS-CoV-2 could be an attractive and effective way to prevent the SARS-CoV-2 infection. 

The crystal structure of the hACE2 receptor and the receptor binding domain (RBD) of the SARS-CoV-2 Spike protein (nCoV-RBD) (PDB: 6M17>) showed that a total of eight residues, namely, Gln24, Asp30, His34, Tyr41, Gln42 in α1 helix, Met82 in α2 helix, and Lys353 and Arg357 in the β3 and β4 linkers, are important for the binding ^9^. The important interactions between the nCoV-RBD with ACE2 are Lys417 (Spike) --Asp30 (hACE2), Tyr453 (Spike) --His34 (hACE2), Gln474 (Spike) --Gln24 (hACE2), Phe486 (Spike) --Met82 (hACE2), Gln498 (Spike) --Tyr41 (hACE2), Thr500 (Spike) --Gln42 (hACE2), and Asn501 (Spike) --Lys353 ^9^.

Peptide-based drugs are a better choice than conventional drugs due to their higher efficiency, lesser molecular weight, and lower toxicity and side effects ^10^. In this regard, antiviral peptides (AVPs), and a subset of antimicrobial peptides (AMPs), are of specific interest due to their higher efficacy in inhibiting viral infection by targeting various stages of the viral life cycle. AVPs can directly invoke innate immune response ^11^ and inhibit viral entry by targeting viral attachment and entry to host cell, and replication, transcription, translation, multiplication, and release inside the host cell ^12, 13^. Previously, several AVPs have been reported to inhibit the SARS-CoV Spike protein or viral entry ^14– 16^.

In this report, using bioinformatics strategies, we attempted to design anti-Spike peptides for SARS-CoV-2 towards motivating potential therapeutics against the SARS-CoV-2 infection.

## Methods

We adopted three strategies to predict potential AVPs against the SARS-CoV-2 Spike protein.

### Strategy I

In the first strategy, we re-analysed the SARS-CoV-2 Spike RBD with hACE2 to identify the key interacting residues in both the proteins. A recent report suggests that the B chain of SARS-CoV-2 Spike protein interacts with the B or D homodomain of hACE2 ^9^. Therefore, in this analysis, we used the individual B chain of SARS-CoV-2 Spike RBD (PDB: 6LZG) and the B chain of hACE2 (PDB: 6M18) to dock with each other using the HADDOCK 2.2 server ^17^, providing active residues of both the proteins as described by Yan *et al.*, (2020) ^9^ and using default parameters. Based on the binding interactions and based on previous reports ^9^, we identified the key interacting residues. In the next step, we designed a number of AVPs based on the interacting hACE2 residues to the RBD of the Spike protein. To design the AVPs, we used simple permutation and combination approach of amino acids, keeping the key interacting amino acids and their positions fixed in the peptide. Binding to SARS-CoV-2 Spike RBD with the designed AVPs was determined by the HPEPDOCK protein-peptide docking server ^18^. For the HPEPDOCK analysis, we used the SARS-CoV-2 Spike RBD (PDB: 6LZG) B chain and the sequence of the peptides in FASTA format, specifying eight binding sites (Lys417, Tyr453, Gln474, Phe486, Gln493, Gln498, Thr500, and Asn501) of Spike-RBD ^9^ and 100 peptide binding mode. The final peptides were selected based on their HPEPDOCK docking energy score, number of binding sites, number and position of selected target residue binding, physiochemical properties, and AVPpred prediction ^13^. More negative binding energy and the number and position of residue binding sites were given more importance in selecting the final peptides (described in *Results*).

### Strategy II

In the second strategy, we screened the available anti-microbial peptides (AMPs) against the SARS-CoV-2 Spike RBD. We used the database of antiviral peptides (AVPdb) ^19^, database of HIV inhibitory peptides (HIPdb) ^12^, Antimicrobial Peptide Database (APD3) ^20^, database of anti-microbial peptides (dbAMP) ^21^, and database of FDA-approved peptide and protein therapeutics (THPdb) ^22^ and screened the peptides against the SARS-CoV-2 Spike RBD. In this process, we collected all the AMP sequences from these databases and then each peptide was docked against the Spike-RBD using the HPEPDOCK protein-peptide docking server ^18^. The process and the parameters of HPEPDOCK docking and selection of peptides were the same as used in first strategy.

### Strategy III

In the third strategy, we adopted a chimeric peptide design approach where the two fragments of two different peptides selected in our previous two approaches are composed in such a way that the resultant peptide can bind to our given target residues in the SARS-CoV-2 Spike RBD. In this approach, we first selected the peptides that bind to any of the three key residues (Phe486, Gln493, and Asn501) of the Spike RBD. Next, we took various lengths of fragments of these peptides (4–15) that interact with the key residues of Spike RBD. Next, we joined these peptides keeping the key residue position fixed to make chimeric peptides of 20–25 amino acids in length. In these peptides, various permutations and combinations of amino acids were made, keeping the key interacting amino acids and their positions fixed. A total of 500 such chimeric peptides were designed and docked with Spike RBD target residues (Lys417, Tyr453, Gln474, Phe486, Gln493, Gln498, Thr500, and Asn501) using the HPEPDOCK server ^18^ as described in the first strategy . The final peptides were selected based on similar criteria as adopted in the first and second strategy.

### Physiochemical analysis of peptides

Antiviral properties of the peptides were predicted using AVPpred ^13^ using its default parameters. The molecular formula, molecular weight, net charge, grand average hydropathy, total hydrophobic ratio, hydrophobicity, and protein-binding potential (Boman index) were calculated using the APD3 antimicrobial peptide calculator and predictor ^20^. The IC _50_ of the peptides was predicted using the AVP-IC _50_Pred server, selecting the RSV/INFV/HSV prediction model ^23^ and other default parameters. Hemolytic potency of peptides was determined using the HemoPI server ^24^ with default parameters, where the values tending towards “0” are unlikely to be hemolytic. ToxinPred ^25^, with default parameters, was used to predict the toxicity (toxic or non-toxic) of the peptides. The final peptides were selected based on their: HPEPDOCK docking energy score (cut-off -120 or less), number of H bonds (>2), number of selected target residue bonds (2-10), number of key target residue bonds (>2), physiochemical properties (parameters acceptable for AMPs, see *Extended data* ^26^), and AVPpred prediction (Yes) ^13^.

## Results and Discussion

### Identification of hACE2 residue-based peptides

In SARS-CoV-2 Spike RBD - hACE2 interaction analysis, similar to Yan *et al.* (2020) ^9^, we found that three stretches of peptides that harbour the active residues of hACE2 interact with Spike RBD. These stretches have amino acid positions: 21-43 (five sites), 78-87 (one site), and 348-361 (two sites). In the Spike RBD, the key interacting amino acid stretches are 480-489 and 490-505. A previous report suggests that among the Spike residues, the most important residues interacting with hACE2 are Phe486, Gln493, and Asn501 ^27^. An in-depth analysis revealed that any peptide that potentially blocks the Spike RBD should bind at least three critical positions of the RBD: (i) Gly485 or Phe486 or Asn487, (ii) Gln493, and (iii) Gln498 or Thr500 or Asn501 among which the Phe486, Gln493, and Asn501 are essential.

**Figure 1.**
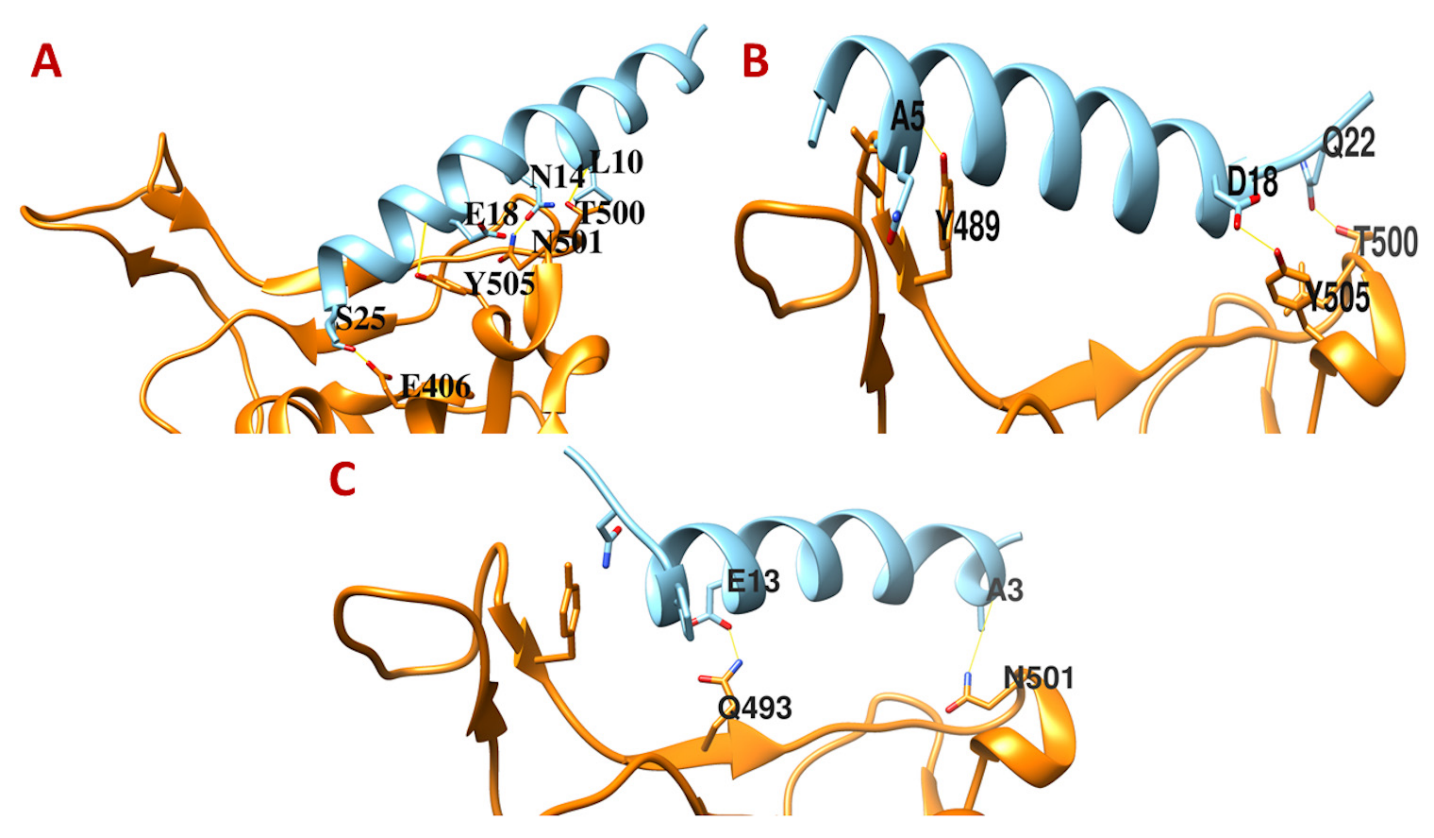
The binding interfaces between SARS-CoV-2 Spike receptor-binding domain with hACE2 derived peptides. ( **A**) AC26, ( **B**) AC23, and ( **C**) AC20.

We designed three peptides from the first stretch of the hACE2 that shows maximum active binding residues with the Spike RBD. A 26 amino acid peptide (AC26) binds to Thr500 and Asn501 of the Spike RBD ( Figure 1A), a 23 amino acid peptide (AC23) binds to Tyr489 and Thr500 of the Spike RBD ( Figure 1B), and a third 20 amino acid peptide (AC20) binds to Gln493 and Asn501 of the Spike RBD ( Figure 1C). All these peptides show acceptable physiochemical properties to be used as therapeutic peptides (see *Extended data* ^26^). However, none of these three peptides are able to block all the key three positions of the Spike RBD. Therefore, these peptides may not be suitable for developing very effective anti-SARS-CoV-2 therapeutics targeting its Spike RBD. However, AC20 and AC23 can be further tested.

### Identification of peptides from antimicrobial peptide databases

We identified seven peptides by screening the five different AMP databases. It is known that SARS-CoV directly interacts with hACE2 through their RBD located in the B chain of the Spike protein ^28, 29^ and the Spike protein sequence of SARS-CoV-2 is highly similar to SARS-CoV ^8^ and SARS-related coronaviruses. In our peptide database analysis, we also observed that the peptides that have been experimentally proven to be effective against SARS-CoV have the potential of being used against the SARS-CoV-2 Spike protein. All of the seven identified peptides (see *Extended data* ^26^) are of 20 amino acids in length and are reported to target the Spike protein of SARS-CoV to exhibit their anti-SARS virus activities ^19^. Although the peptides show four to 11 “H” bonds and form between two to four bonds with our eight given target residues, most of these peptides do not bind to all the three positions ((i) Gly485 or Phe486 or Asn487, (ii) Gln493, and (iii) Gln498 or Thr500 or Asn501) to effectively block the Spike RBD.

**Figure 2.**
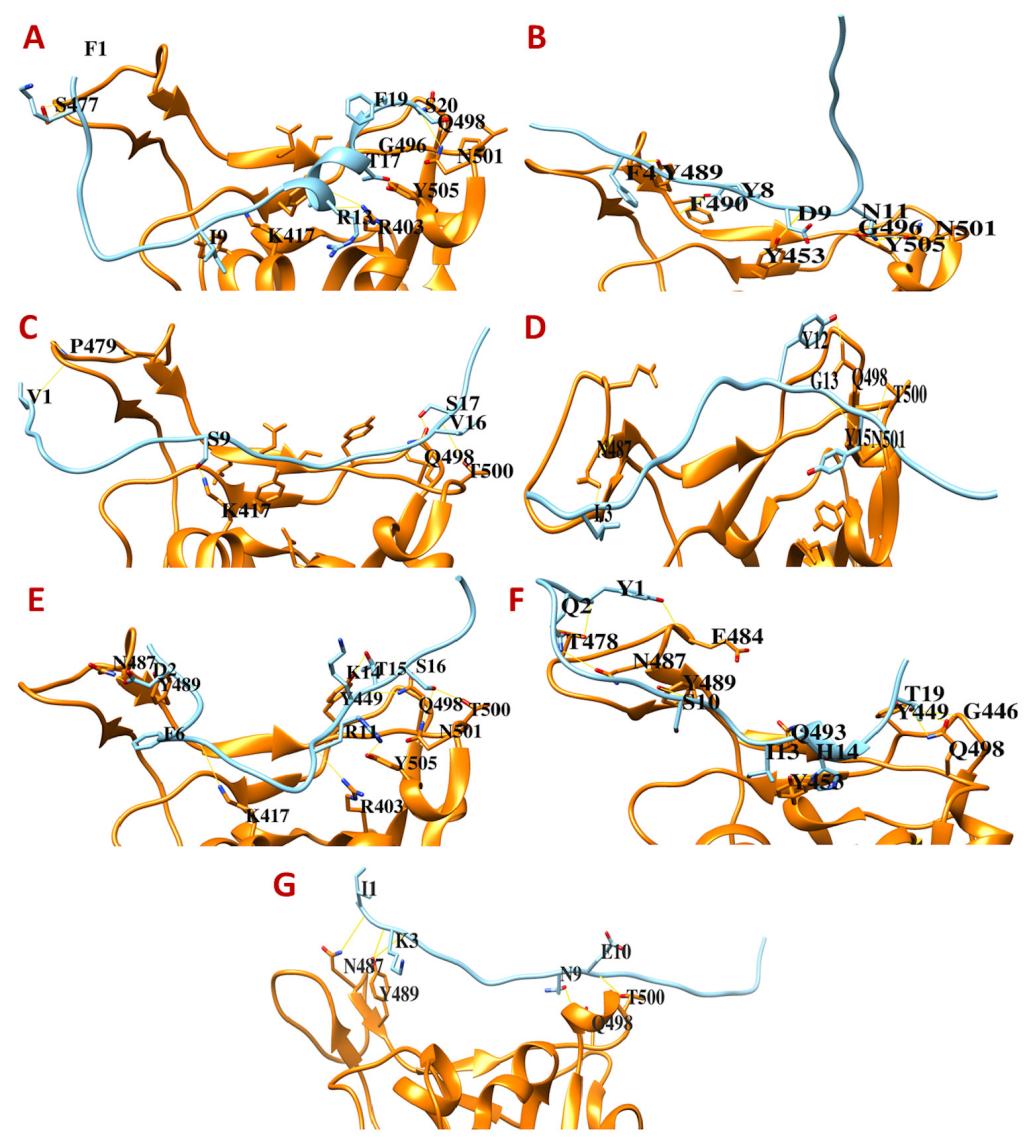
The binding interfaces between SARS-CoV-2 Spike receptor-binding domain with peptides screened from the antimicrobial peptide databases. ( **A**) DBP1, ( **B**) DBP2, ( **C**) DBP3, ( **D**) DBP4, ( **E**) DBP5, ( **F**) DBP6, and ( **G**) DBP7.

The DBP1, DBP2, and DBP3 peptides bind to only the third position (Gln498 and Asn501) of the Spike RBD without binding to the other two sites ( Figure 2A–C; *Extended data* ^26^). DBP4, DBP5, and DBP7 interact with the first (Gly485 or Phe486 or Asn487) and third (Gln498 and Asn501) sites of the Spike RBD without binding to the middle or the second site (Gln493) ( Figure 2D, E, G; *Extended data* ^26^). DBP6 binds to all the three sites within the range of the target residues but does not interact with the key residues of the first and third sites ( Figure 2F; *Extended data* ^26^). DBP6 is also predicted to be an antiviral peptide by AVPpred ^13^. Therefore, DBP6 could be a potential peptide to be tested for SARS-CoV-2 Spike protein-based drug development.

### Chimeric peptides against SARS-CoV-2 Spike RBD

**Figure 3.**
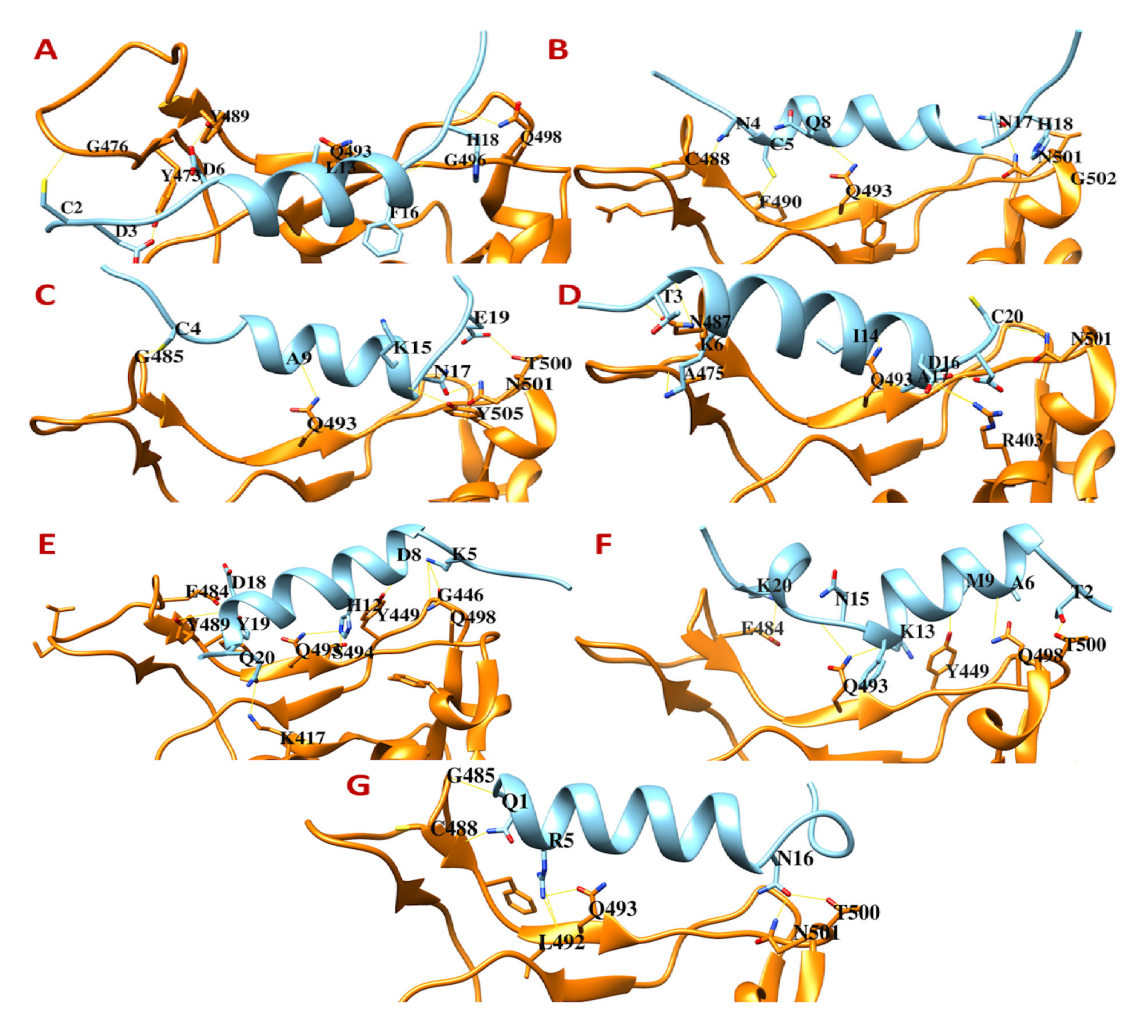
The binding interfaces between SARS-CoV-2 Spike receptor-binding domain with designed chimeric peptides. ( **A**) cnCoVP-1, ( **B**) cnCoVP-2, ( **C**) cnCoVP-3, ( **D**) cnCoVP-4, ( **E**) cnCoVP-5, ( **F**) cnCoVP-6, and ( **G**) cnCoVP-7.

Out of 500 chimeric peptides generated, only seven were selected for final analysis. All these peptides are non-hemolytic, non-toxic, and meet all the criteria of a therapeutic peptide (see *Extended data* ^26^). Among these seven peptides, cnCoVP-3, cnCoVP-4, and cnCoVP-7 interact with all the three sites and two key residues of the second (Gln493) and third (Asn501) sites. However, these peptides bind one amino acid apart from the key residue (Phe486) in the first site and potentially block the access of SARS-CoV-2 Spike Phe486 to the hACE2 ( Figure 3C, D, G; *Extended data* ^26^). AVPpred ^13^ also predicted cnCoVP-4 to be an antiviral peptide. Therefore, these three peptides could be selected for further *in vitro* and *in vivo* testing. 

Although the chimeric peptides cnCoVP-2, cnCoVP-5, and cnCoVP-6 interact with all the three sites, they do not interact with the key residue (Phe486) or the immediate to key residue of the first site. Instead they bind a residue that is two to three amino acids apart from the key residue (Phe486) ( Figure 3B, E, F; *Extended data* ^26^). Therefore, these three peptides may not block the first site of the Spike RBD in interacting with hACE2. However, they should also be synthesized and tested for their *in vitro* effects. The last peptide, cnCoVP-1, was found to interact with all the three sites; however, it only interacts with the key residue (Gln493) of the second site. In the other two sites, it interacts at position (Tyr489) of the first site and (Gln498) of the third site ( Figure 3A; *Extended data* ^26^). Although Gln498 is a key residue of the third site Tyr489, it is not an interacting residue in the original interaction between SARS-CoV-2 Spike RBD and hACE2. Thus, this peptide may partially block the access of SARS-CoV-2 Spike RBD to hACE2 and needs further *in vitro* and *in vivo* testing and validation.

## Conclusions

In this article, we screened and designed several peptides that may potentially block the interaction between SARS-CoV-2 Spike RBD and hACE2. Ten peptides (AC20, AC23, DBP6, and cnCoVP-1- cnCoVP-7) have very high potential to achieve this interaction, indicating that these peptides could be attractive therapeutics against SARS-CoV-2. However, peptide synthesis, *in vitro*, and *in vivo* experiments are required to evaluate and ensure their potential therapeutic efficacy. 

## Data availability

### Source data

B chain of SARS-CoV-2 Spike RBD from PDB, Accession number 6LZG: https://identifiers.org/rcsb/pdb:6LZG

B chain of hACE2 from PDB, Accession number 6M18: https://identifiers.org/rcsb/pdb:6M18

## Extended data

Harvard Dataverse: Potential chimeric peptides to block the SARS-CoV-2 Spike RBD. https://doi.org/10.7910/DVN/WSDRTU ^26^

This project contains the following extended data:

- MS_cnCoVP_Supplementary_Table-S1_F1000.xlsx (detailed physiochemical and docking properties and AVPpred prediction of each peptide)

Data are available under the terms of the Creative Commons Zero "No rights reserved" data waiver (CC0 1.0 Public domain dedication).
