# Supplementary material for: Potential chimeric peptides to block the SARS-CoV-2 spike receptor-binding domain
Source: F1000Res. 2020 Jun 9;9:576. [Version 1] doi: 10.12688/f1000research.24074.1 (PMC7411520; doi:10.12688/f1000research.24074.1)
Supplement: Peer review history

# Peer review: Reviewer response for version 1

**DOI:** 10.5256/f1000research.26557.r68734

**Published:** 2020-08-24

**Authors:** Amjad Ali

**Affiliations:** 1 Atta-ur-Rahman School of Applied Biosciences (ASAB), National University of Sciences and Technology (NUST), Islamabad, Pakistan

The study manuscript “ *Potential chimeric peptides to block the SARS-CoV-2 spike receptor-binding domain*” by Barh *et al.*, focuses on chimeric peptides to potentially block the SARS-CoV-2 receptor binding domain; the work is interesting and well designed and written. I have few minor comments and suggestions if they could be considered by the authors for improvement and clarification.

 As mentioned *“SARS-CoV binds to the human angiotensin-converting enzyme 2 (hACE2) receptor through its Spike protein (S) to enter into the host cell* *^6^* *, and it is now reported that SARS-CoV-2 also binds to ACE2 to transmit its genetic material to human cells* *^7^* *^–^* *^9^* *”*

 In these lines the authors stated that SARS-CoV-2 also binds to ACE2, I think the authors intended to write hACE2 (as stated earlier). The abbreviation is inconsistent in the whole manuscript. Please have a look.

**Comment:** The authors stated that AVP are a better choice in catering viral infection, but as they are peptides, they are prone to be attacked by proteases. How to overcome this issue?

**Comment:** Do these AVPs have a role in generating memory response? If the AVPs directly evoke innate immune response there is a possibility the PAMPs present on these can be recognized by PRRs which may lead to degradation.

**Comment:** A study similar to Strategy 1 has been conducted by Ibrahim *et al. (*2020 ^1^) to predict binding residues between RBD and hACE2. Does your study validate those findings? Are there any differences?

**Comment:** Which tool or database was used to design AVPs in strategy 1?

**Comment:** As the authors discussed the hemolytic potential of chimeric peptides, were peptides designed using Strategy 1 and Strategy 2 also fulfilling the criteria of therapeutic potential? Also, among the ten peptides mentioned in the conclusion which of them are best? Is it possible to give a brief comparison of these 10 peptides?

Is the work clearly and accurately presented and does it cite the current literature?

Yes

If applicable, is the statistical analysis and its interpretation appropriate?

Not applicable

Are all the source data underlying the results available to ensure full reproducibility?

Partly

Is the study design appropriate and is the work technically sound?

Yes

Are the conclusions drawn adequately supported by the results?

Yes

Are sufficient details of methods and analysis provided to allow replication by others?

Partly

Reviewer Expertise:

NA

I confirm that I have read this submission and believe that I have an appropriate level of expertise to confirm that it is of an acceptable scientific standard, however I have significant reservations, as outlined above.

## References

[1] : COVID-19 spike-host cell receptor GRP78 binding site prediction. *J Infect*.80(5) : 10.1016/j.jinf.2020.02.026 554-562 10.1016/j.jinf.2020.02.026 32169481PMC7102553

# Peer review: Reviewer response for version 1

**DOI:** 10.5256/f1000research.26557.r68731

**Published:** 2020-08-06

**Authors:** Shailendra Dwivedi

**Affiliations:** 1 Department of Biochemistry, All India Institute of Medical Sciences, Gorakhpur, Uttar Pradesh, India

The overall article is well written and has properly compiled the report on "Potential chimeric peptides to block the SARS-CoV-2 spike receptor-binding domain" which is oriented on the present pandemic of COVID-19. The authors have screened and designed many peptides that can potentially block the interaction between SARS-CoV-2 Spike RBD and hACE2. Thus, the authors concluded that ten peptides (AC20, AC23, DBP6, and cnCoVP-1- cnCoVP-7) have a very high potential of interaction, indicating that these peptides could be attractive therapeutics against SARS-CoV-2. 

 The authors have used proper methodologies to conclude the report, so it can be accepted for indexing.

Is the work clearly and accurately presented and does it cite the current literature?

Yes

If applicable, is the statistical analysis and its interpretation appropriate?

Yes

Are all the source data underlying the results available to ensure full reproducibility?

Yes

Is the study design appropriate and is the work technically sound?

Yes

Are the conclusions drawn adequately supported by the results?

Yes

Are sufficient details of methods and analysis provided to allow replication by others?

Yes

Reviewer Expertise:

Molecular Biology, Cancer Stem Cell, Epigenetics, Nutrigenomics, Cancer Biology

I confirm that I have read this submission and believe that I have an appropriate level of expertise to confirm that it is of an acceptable scientific standard.

# Peer review: Reviewer response for version 1

**DOI:** 10.5256/f1000research.26557.r64504

**Published:** 2020-06-29

**Authors:** Candan Hizel

**Affiliations:** 1 OPTI-THERA Inc./Medpharmgene, Montreal, QC, Canada

The coronavirus disease 2019 (COVID-19) pandemic has created a worldwide crisis and inspired an urgent search for prevention and treatment of severe acute respiratory syndrome coronavirus 2 (SARS-CoV-2) infection.

 Barh and his colleagues describe in this article the important role of immuno-informatics by using molecular modeling and docking for chimeric peptide design anti-Spike peptides for SARS-CoV-2 potential therapeutics against the covid-19 infection. The introduction provides a good, generalized background of the topic that quickly gives the reader an appreciation of the applications of the technique. “A large global effort is required to stop viral epidemics and pandemics, and these manuscript insights may lead to identifying novel ways to stop infection.” To this end, this manuscript highlights clearly the potential opportunities several peptides blocking the interaction between SARS-CoV-2 Spike RBD and hACE2 as probable application in clinical practice.

 Overall the topic and the data are very interesting. The manuscript is very well written, well organized, easy to follow, and appropriate in scope and method. Certainly this study will contribute much to the literature.

Is the work clearly and accurately presented and does it cite the current literature?

Yes

If applicable, is the statistical analysis and its interpretation appropriate?

Yes

Are all the source data underlying the results available to ensure full reproducibility?

Yes

Is the study design appropriate and is the work technically sound?

Yes

Are the conclusions drawn adequately supported by the results?

Yes

Are sufficient details of methods and analysis provided to allow replication by others?

Yes

Reviewer Expertise:

Pharmacogenomics

I confirm that I have read this submission and believe that I have an appropriate level of expertise to confirm that it is of an acceptable scientific standard.

# Response

**Published:** 2020-06-30

**Authors:** Debmalya Barh

**Affiliations:** IIOAB, India

Thank you for your critical review and approval.
